# Supplementary material for: Loss of the Bardet-Biedl protein Bbs1 alters photoreceptor outer segment protein and lipid composition
Source: Nat Commun. 2022 Mar 11;13:1282. doi: 10.1038/s41467-022-28982-6 (PMC8917222; doi:10.1038/s41467-022-28982-6)
Supplement: Supplementary file 1 — Supplementary Information [file 41467_2022_28982_MOESM1_ESM.pdf]

Supplementary data

Loss of the Bardet-Biedl protein Bbs1 alters photoreceptor outer segment protein and lipid composition

Markus Masek[1,2§], Christelle Etard[3§], Claudia Hofmann[1,2], Andreas J. Hülsmeier[4], Jingjing Zang[2], Masanari Takamiya[3], Matthias Gesemann[2], Stephan C.F. Neuhauss[2], Thorsten Hornemann[4], Uwe Strähle[3,5,#], Ruxandra Bachmann-Gagescu[1,2#,*]

[1]*Institute of Medical Genetics, University of Zurich, Zurich, Switzerland*
[2]*Department of Molecular Life Sciences, University of Zurich, Zurich, Switzerland*
[3] *Institute of Biological and Chemical Systems (IBCS-BIP), Karlsruhe Institute of Technology, Karlsruhe, Germany*
[4]*Institute of Clinical Chemistry, University Hospital Zurich, Switzerland*
[5]*Center of Organismal Studies, University of Heidelberg, Germany*

Supplementary Fig. 1: **Synteny of the zebrafish *bbs1* locus**

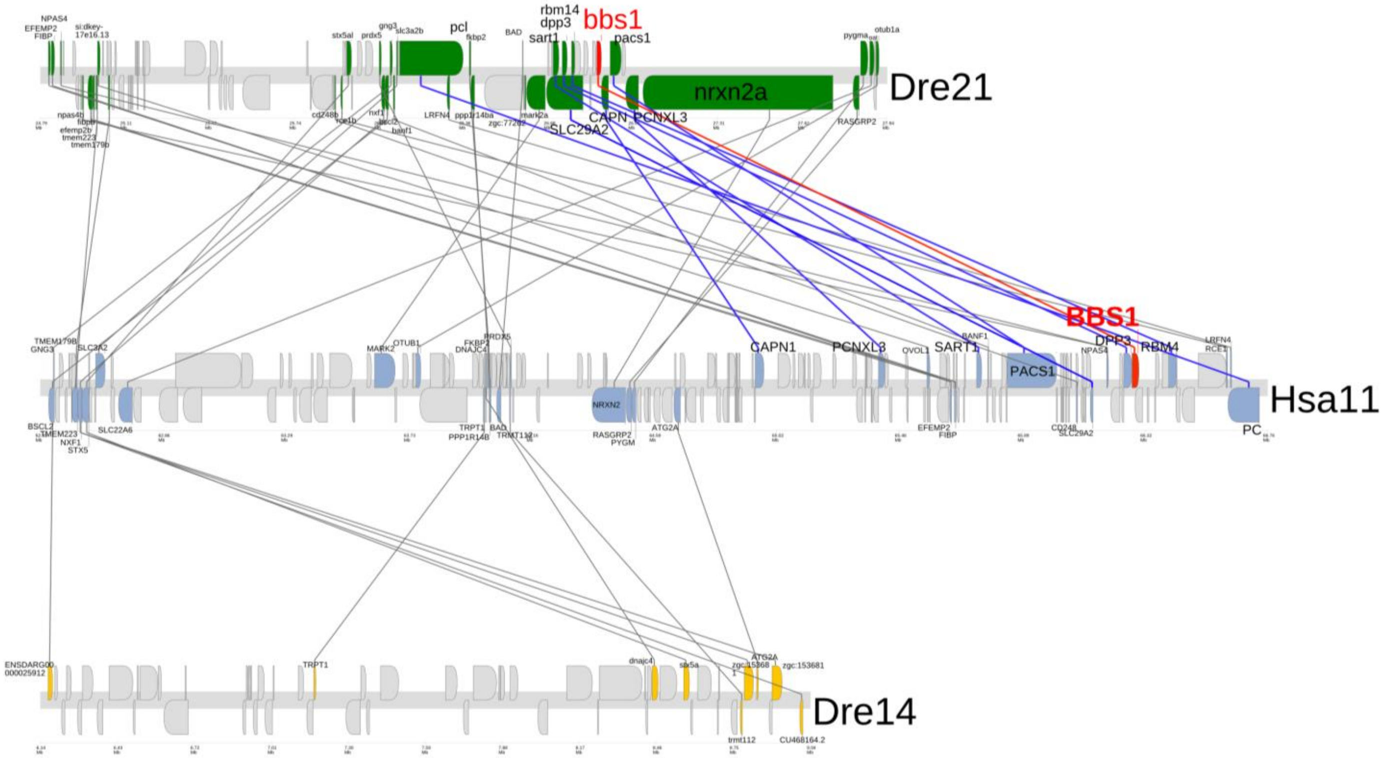

Genes flanking *BBS1* located on human chromosome 11 are found on zebrafish chromosomes 21 and 14. BBS1 genes are highlighted in red. The localization within the chromosome is given in the scale bars below the chromosomes. Orthologous genes between human (blue) and corresponding genes on zebrafish chromosomes 21 (green) and 14 (yellow), are depicted. The grey lines linking corresponding genes indicate the relative position of the genes on the chromosome and point out the single zebrafish orthologue. Orthologous genes directly flanking the human BBS1 locus are highlighted by dark blue lines. Note that most of these genes are found around the *bbs1* locus of zebrafish chromosome 21. *Dre* Danio rerio, *Hsa* Homo sapiens.

Supplementary Fig. 2: **Protein homology between human and zebrafish Bbs1**

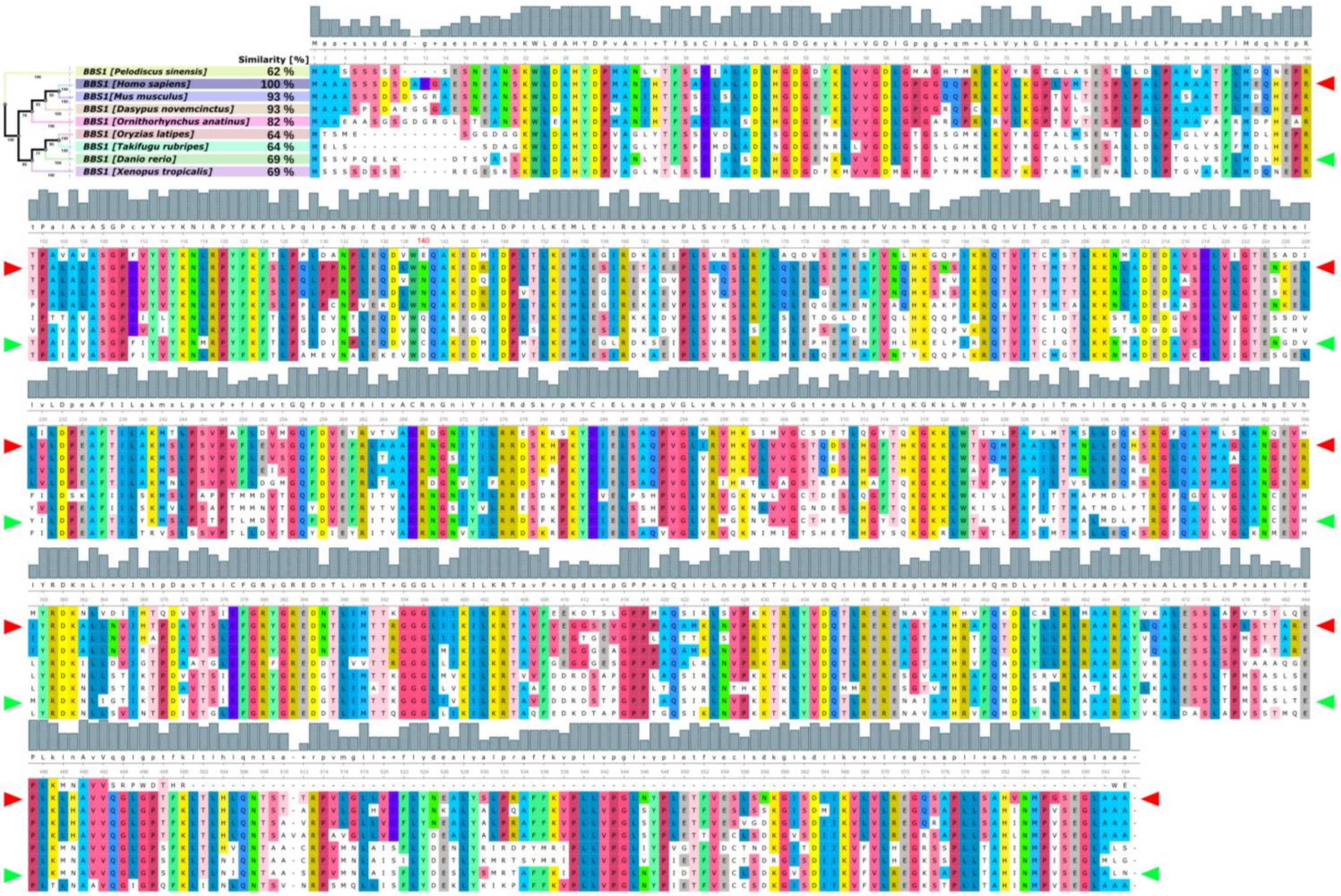

Amino acid sequences of the following species were aligned using Unipro UGENE (vers. 36.0), configured for high accuracy: Human *(Homo sapiens)*, mouse *(Mus musculus)*, zebrafish *(Danio rerio)*, torafugu *(Takifugu rubripes)*, medaka *(Oryzias latipes), armadillo (Dasypus novemcinctus), Chinese softshell turtle (Pelodiscus sinensis) platypus (Ornithorhynchus anatinus) and xenopus (Xenopus tropicalis).* Conservation is displayed as a bar graph (grey boxes) and the most conserved amino acid is the consensus (above sequence alignment at a given position). Conservation of the amino acid sequence between human and zebrafish Bbs1 is 69%. The red arrowhead points to the human sequence and the green arrowhead to the zebrafish sequence.

Supplementary Fig. 3: **No evidence for residual Bbs1 function in the *bbs1*^{k742} allele**

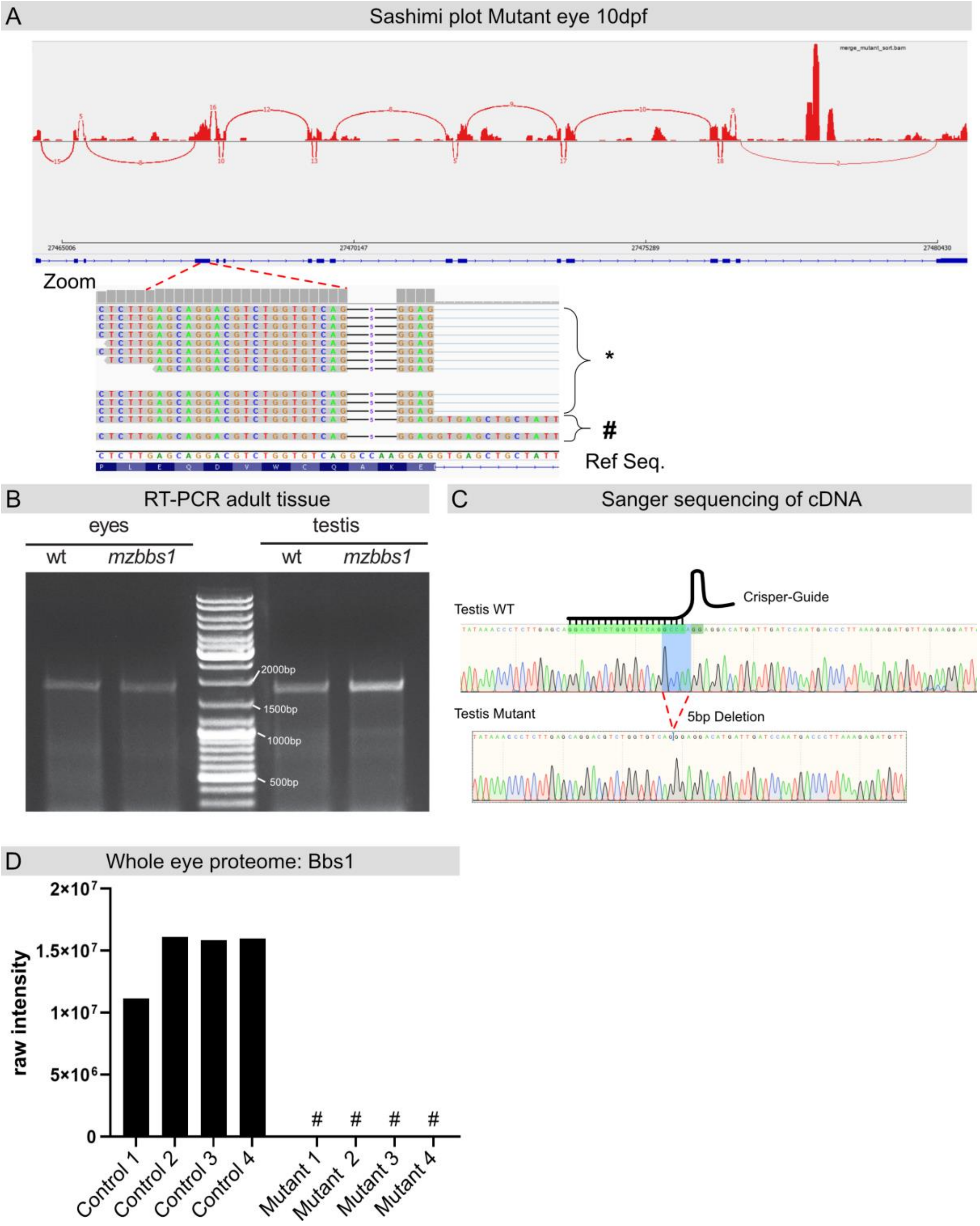

(A) Sashimi plot from the 10dpf mutant eye-specific RNAseq experiment showing reads spanning across exon-exon junctions. All the reads include the 5bp deletion and there is no evidence for skipping of exon 4. * Reads continuing (represented by the fine line on the right) into the the next exon 5. # reads that include intron 4, which likely represent pre-spliced mRNA; in any case, inclusion of intron 4 would result in mutliple stop codons. *Ref Seq* Reference Sequence. (**B**) Amplification of *bbs1* from cDNA generated from

whole eye and testis of adult (12 month old) control and *bbs1*-/- fish. In both tissues only one band of full length *bbs1* without alternative splice forms was identified. To rule out low levels of alternative isoforms, an endpoint PCR was performed, explaining why the band in mutant does not appear fainter than the control band. To rule out small base pair changes which could restore the original reading frame, the amplicon was sequenced. This experiment was performed with n= 2 animals per condition. (**C**) Sanger sequencing revealed that no additional small indel is present in mutants besides the expected 5bp deletion. The only mRNA present thus includes the 5bp deletion leading to a premature stop codon. (**D**) Bar plot showing the summed peptide intensities for Bbs1 from LC -MS/MS using an inclusion list on adult (9 month old) whole eye lysates. Note consistent presence of signals in all 4 controls and absence of even a single signal in mutants, supporting lack of Bbs1 protein. Sample size (n=4 Control, n=4 Mutants), (#) defines a complete absence of any signal. Raw data are provided in supplementary data 1.

Supplementary Fig. 4: Ciliogenesis is unaffected in *bbs1-/-* zebrafish larvae

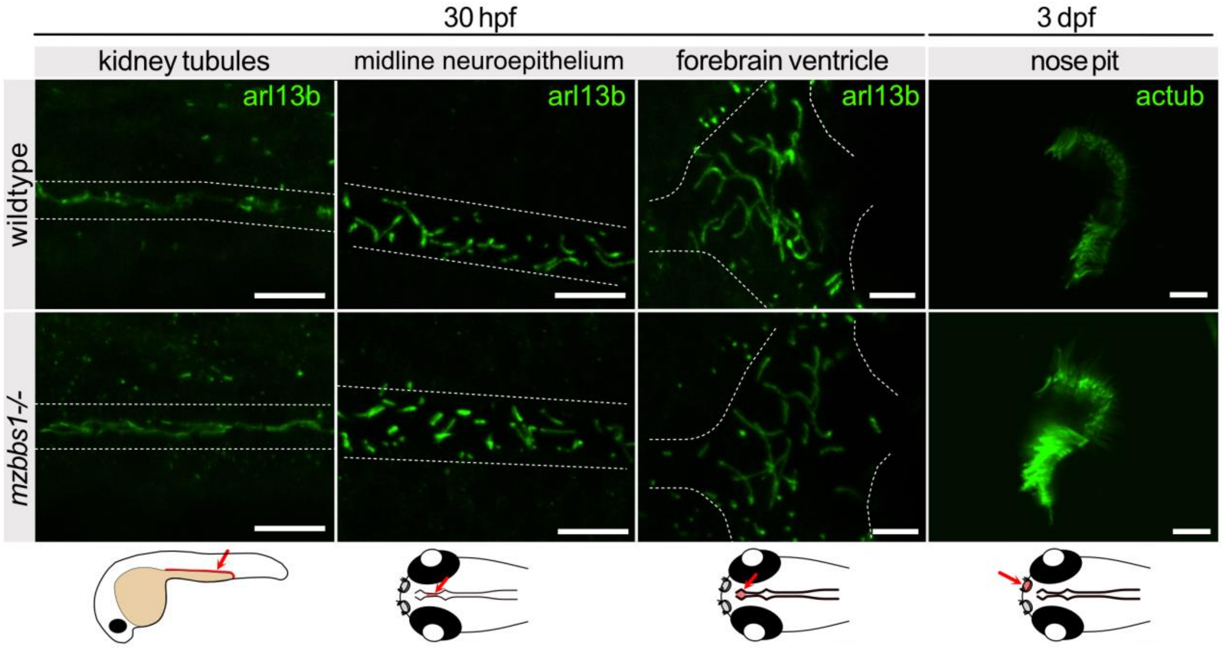

Whole mount immunostaining of various ciliated tissues including kidney tubules, midline neuroepithelium, forebrain ventricle cilia at 30 hours post fertilization (hpf) and nose pit cilia at 3 days post fertilization (dpf). Cilia are labeled using anti-Arl13b (green) or anti-acetylated tubulin (actub) antibodies (green) as indicated. No differences in abundance or morphology of cilia were noted between *mzbbs1* mutants (bottom) and their sibling controls (top). All images are dorsal views with rostral to the left and caudal to the right. The schematics at the bottom are for orientation. Scale bars: 10µm

Supplementary Fig. 5: Lack of opsin mislocalization despite progressive retinal dystrophy

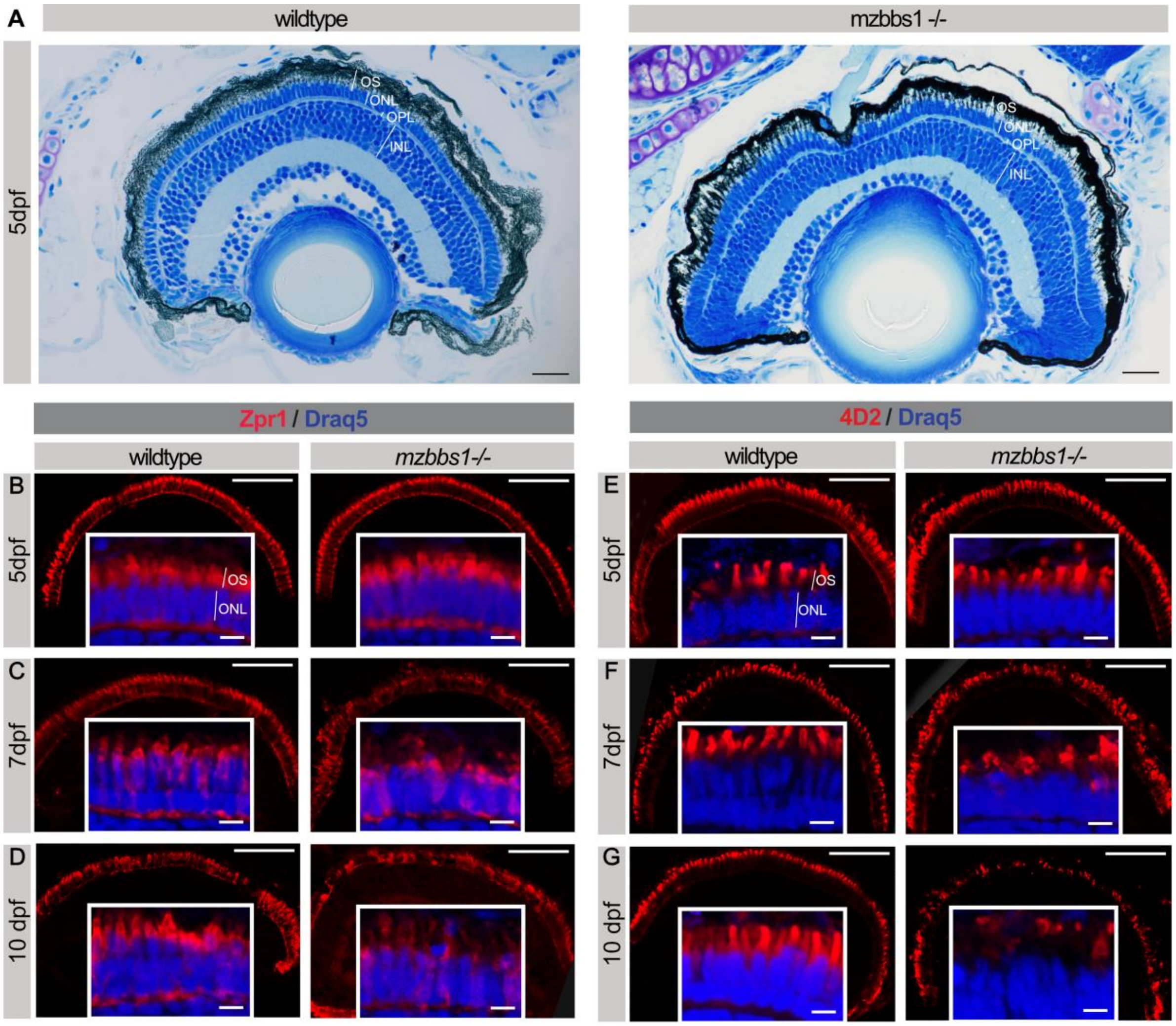

(**A**) Semi-thin plastic section of *mzbbs1*$^{-/-}$ mutant and control eyes stained with Richardson solution at 5 dpf are indistinguishable from each other. Retina lamination is unaffected by the mutation with respect to the overall morphology. The INL, ONL and OPL thickness in mutants are comparable to controls. (**B-D**) Immunohistochemistry staining of red-green cones using zpr1 (red) reveals no morphological changes at 5 dpf (**B**). Starting at 7 dpf (**C**) slight morphological abnormalities are observed that progress over time and at 10 dpf (**D**) the OS layer is substantially thinner and disorganized. (**E-G**) Immunohistochemistry of opsins using the 4D2 antibody (red) shows no opsin mislocalization at 5 dpf (**E**), 7 dpf (**F**) or 10 dpf (**G**) despite the severe morphological changes. Draq5 (blue) was used to counter stain the nuclei. Scale bars: (A): 20 µm, (B-G): 50 µm, inserts: 10 µm; Abbreviations: *OS*, outer segment; *ONL*, outer nuclear layer; *OPL*, outer plexiform layer; *INL*, inner nuclear layer.

Supplementary Fig. 6: **Increased photoreceptor cell death in *mzbbs1*$^{-/-}$ mutants at 10 dpf**

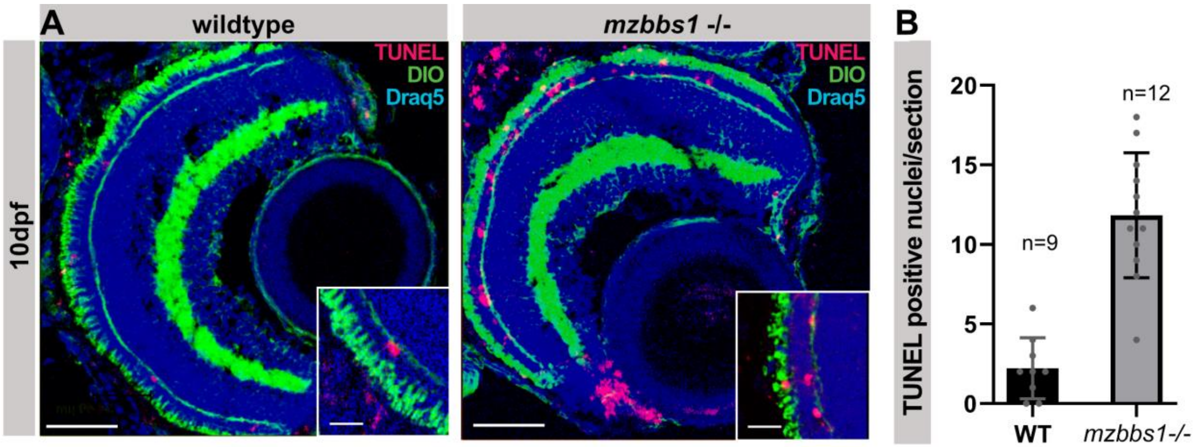

Apoptotic cells are labeled using the ApopTag® Red In Situ Apoptosis Detection Kit in control and *mzbbs1* mutants **(A)**. Quantification of apoptotic cells per section is shown in **(B).** Error bars mark the standard deviation around the mean. Sample size (Ctrl, n=9 sections of 4 larvae; Mutant: n=12 sections of 6 larvae). Scale Bar: (**A**) 47 µm, insert: 12 µm.

Supplementary Fig. 7: Slowly progressive retinal dystrophy in zygotic *bbs1* mutants

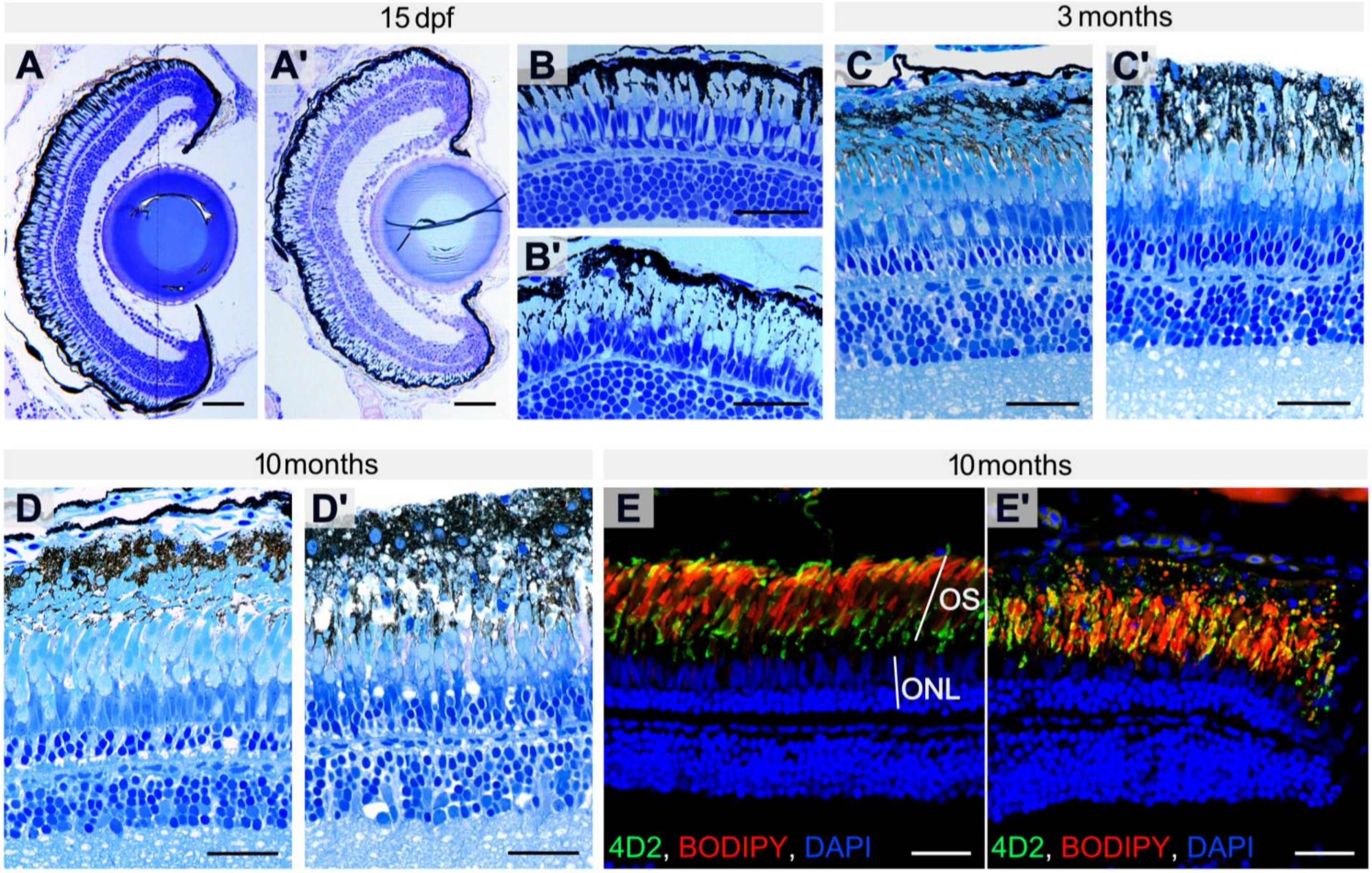

(**A-D'**) Semi-thin plastic sections of *zgbbs1*$^{-/-}$ mutants at 15 dpf (**A', B'**), 3 months (**C'**) and 10 months (**D'**) compared to controls (**A-D**) show a slow progression of retinal dystrophy. At 3 months post fertilization (mpf) some healthy-looking outer segments are still present in mutant retinas (**C'**). At 10 mpf the mutant OSs layer is severely disrupted (**D'**) and looks degenerated compared to control (**D**). Nuclei are observed within the mutant OS layer at 10 months, suggesting an invasion of microglia or abnormal RPE (**D'**). (**E, E'**) Immunostaining of opsins using 4D2 (green) reveals no opsin mislocalization in the IS or ONL in the retina of 10 mpf mutants (**E'**). The sections are co-stained with the membrane specific dye BODIPY to label OSs (red) and DAPI for nuclei (blue). Scale bar: (A, A'): 50 µm; (B-E'): 30 µm; Abbreviations: *OS* outer segment, *ONL* Outer nuclear layer

Supplementary Fig. 8: Zygotic *bbs1* mutants show a slow decrease in visual function

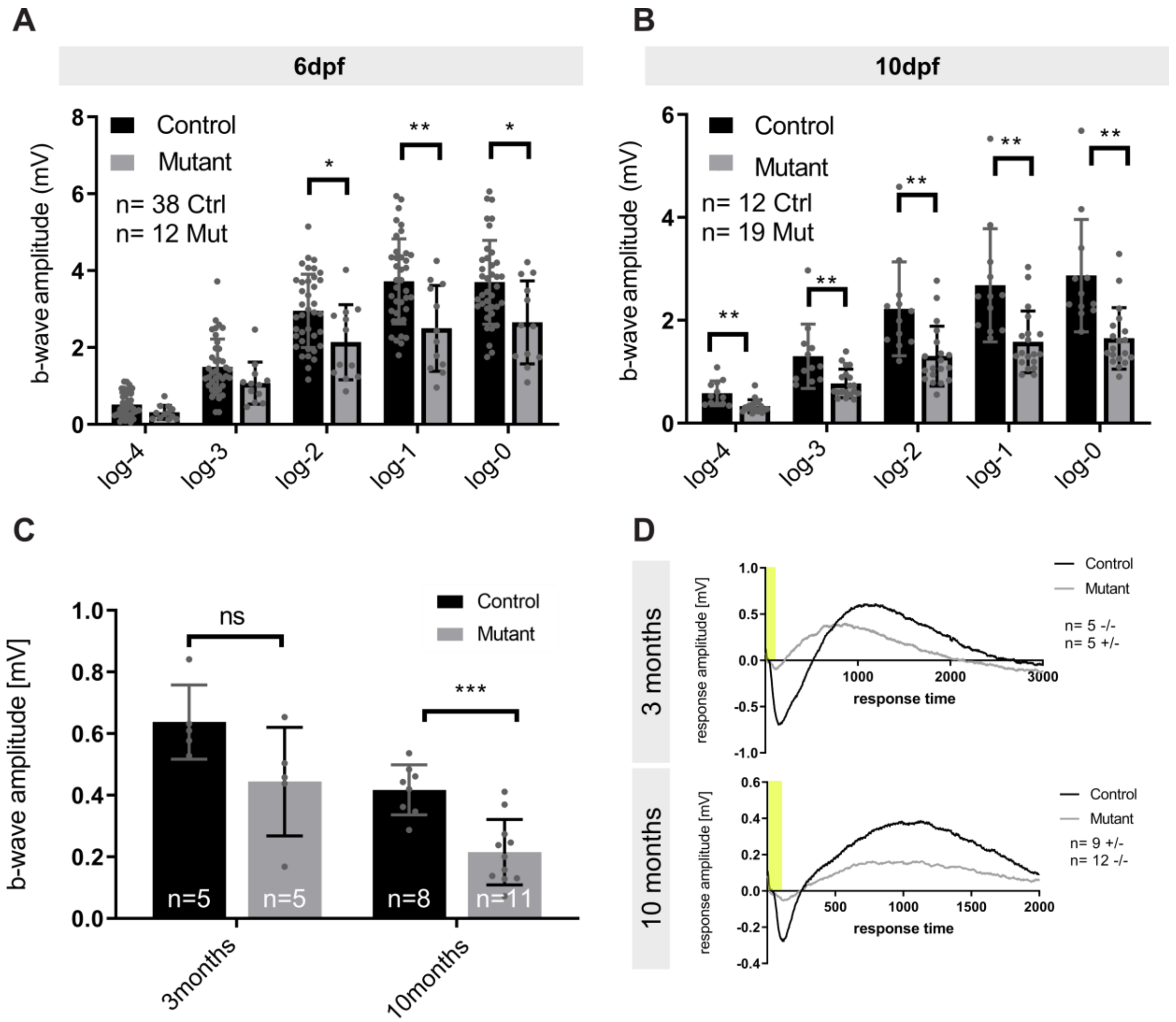

The functional phenotype of *zgbb1* mutants was assessed using ERG in larvae at 6 and 10 dpf as well as in adults at 3 and 10 months post fertilization. (**A-B**) Bar plots of the maximum b-wave amplitude by electroretinography (ERG) shows a significantly decreased response to light in *zgbbs1*$^{-/-}$ mutants for high light intensities (log-0 to log-2) at 6 dpf (**A**) and for all light intensities (log-0 to log-4) at 10 dpf (**B**). Unpaired two-tailed multiple T-test; Sig: ns= FDR(q-value)>0.05; *= FDR(q-value)<0.05; **= FDR(q-value)<0.01; Error bars show standard deviation around the mean. Each dot in the plots represents one animal (n= number of animals). For more detailed statistics, please see **Supplementary data 6**. (**C**) Bar plot of the ERG response of adult fish at 3 and 10 months, at the highest light intensity (log-0 corresponds to 24'000µW/cm^2). Unpaired two-tailed multiple T-test, Holm-Sidàk adjusted; Sig: ns=adj. P-value=0.077, ***= adj. P-value <0.001, Sample size (n=5 WT, n=5 Mut eyes at 3 months & n=8 WT, n=11 Mut eyes at 10 months); Error bars show standard deviation around the mean. (**D**) Average ERG response curve after a 100 ms light flash (yellow box) for wt (black) and mutant (grey) at 3 months in top panel and 10 months in bottom panel. The lack of statistical significance at 3 months is likely explained by the technical difficulty of measuring ERGs in adult zebrafish and the limited number of animals that can be raised. The clearly reduced average curve and the altered shape of the curve at this stage (**D**) support a visual deficit at 3 months already. Abbreviations: *ERG* Electroretinography. Source data are provided as a Source Data file.

Supplementary Fig. 9: **Expression levels of BBSome components in the adult retina**

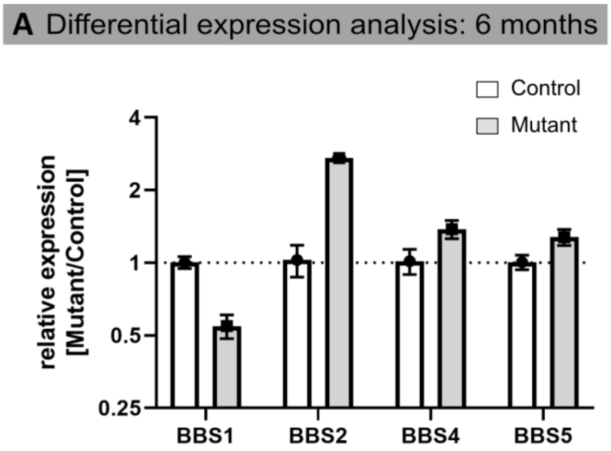

B Primer table

Name	Primer	Sequence 5'-3'
BBS1_Frw	For.	CTC CTC ATG CAT TGC TCT TTC AG
BBS1_Rev	Rev.	CAC GAT ACA CCT TCA GCT TCA TG
BBS2_Frw	For.	AGA TGG CAG AAA ATG AGA CAG TCA C
BBS2_Rev	Rev.	TTT GAC TTG ATC CTC CAG TAG CG
BBS4_Frw	For.	GCT GTG TAT GTG CAA GCT CTG
BBS4_Rev	Rev.	TCC CTA ACA GGA AAA GCG ATC G
BBS5_Frw	For.	CGG AGA TAG AGG GAG ACT TCT GG
BBS5_Rev	Rev.	GTA TCC CAC AGA CAG GTT GAC TC
g6pd_Frw	For.	CTG GAC CTG ACC TAC CAT AGC AG
g6pd_Rev	Rev.	AGG CTT CCC TCA ACT CAT CAC TG

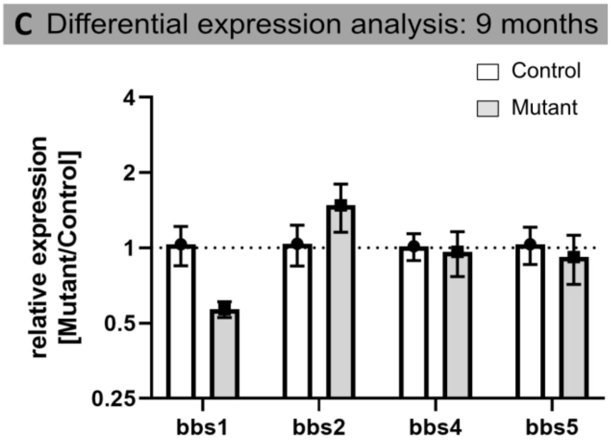

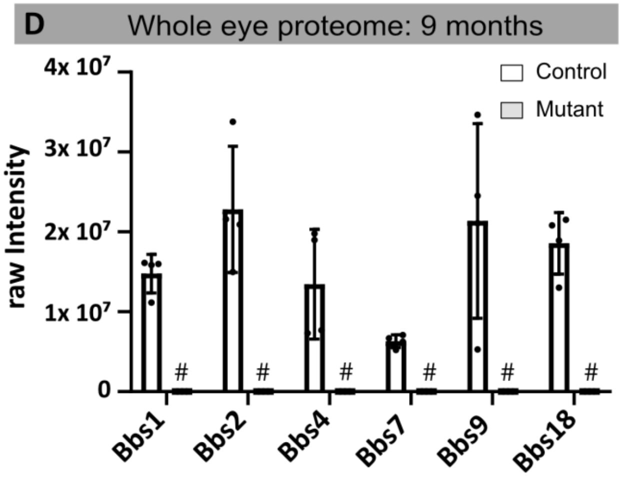

The mRNA expression levels of BBSome components in the whole eye of 6 month old **(A)** and 9 month old **(C)** control and *zgbbs1*$^{-/-}$ whole eyes are shown as bar plots of the mean with SEM. Biological replicates were normalized to the expression of the housekeeping gene *g6pd* and the relative mRNA expression level was calculated using the mean control expression levels. Sample size (A) n=3 Control & n=3 Mutant, (C) n=3 Control & n=4 Mutant. Note that at both time points *bbs1* mRNA levels are decreased while the other tested BBSome subunits were either unchanged or slightly upregulated. (**B**) Primer sequences used for the real time PCR experiment. (**D**) Bar plots showing mean and standard deviation of the raw intensities from LC-MS/MS (with inclusion list) of BBSome components on whole eye lysates, using the second eye of the same 9 month-old adult fish as for the qPCR experiment in (C). The plot shows the sum of the raw intensities of all identified unique peptides corresponding to a given protein. Note that in all control samples BBSome components were found, whereas these were absent from the mutant lysates (no signal indicated by #). Of note, some BBSome components (Bbs5 and Bbs8) were not identified even in controls, which is likely explained by technical limitations (for example due to poor ionization efficiency). Sample size (D) n=4 Control & n=4 Mutant samples.

Supplementary Fig. 10: Proteomic analysis of samples enriched for OSs - Quality Controls

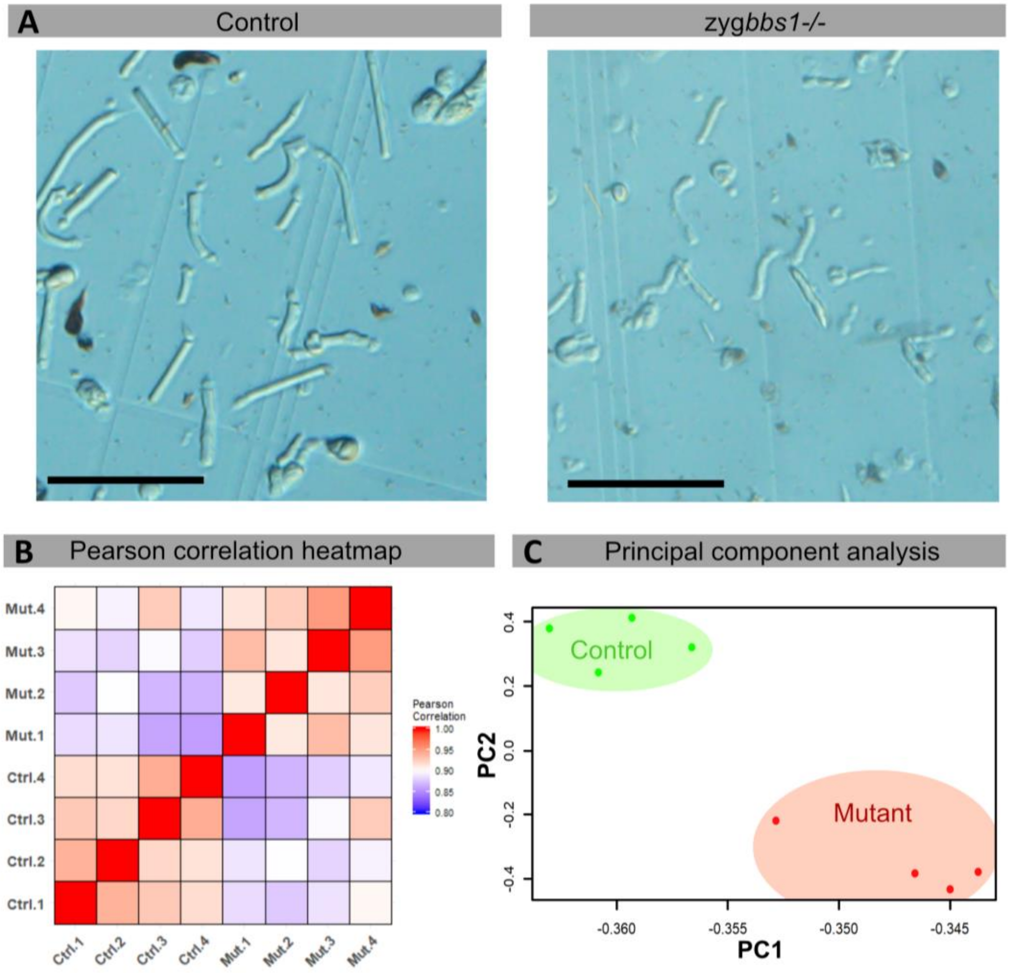

(**A**) Representative image of the samples after mechanical isolation of OSs from 5 month old control (left) and mutant (right) eyes *before* purification through a sucrose gradient. Overall, the OSs in the mutants appear to be smaller and some look deformed while others have a normal appearance. In both images slight contamination of pigmented RPE and other cell fragments is still present. Subsequent sucrose gradient purification was then performed to further clean the sample based on density differences (no imaging was possible after the sucrose gradient purification). Scale bar: 50µm. (**B**) Pearson correlation comparing the proteomics results between all measured samples reveals that the "within condition" correlation (controls compared to each other and mutants compared to each other) is larger than "between conditions" (controls versus mutants). Overall, all correlations are high (≥0.8) indicating that no large differences in the *overall* protein landscape exist between samples. (**C**) Nevertheless, a clear separation and clustering of samples according to condition (control or mutant) using components 1 and 2 of the principal component analysis is visible, indicating distinct protein signatures between mutant and control.

Supplementary Fig. 11: Proteomic analysis on samples enriched for isolated OSs

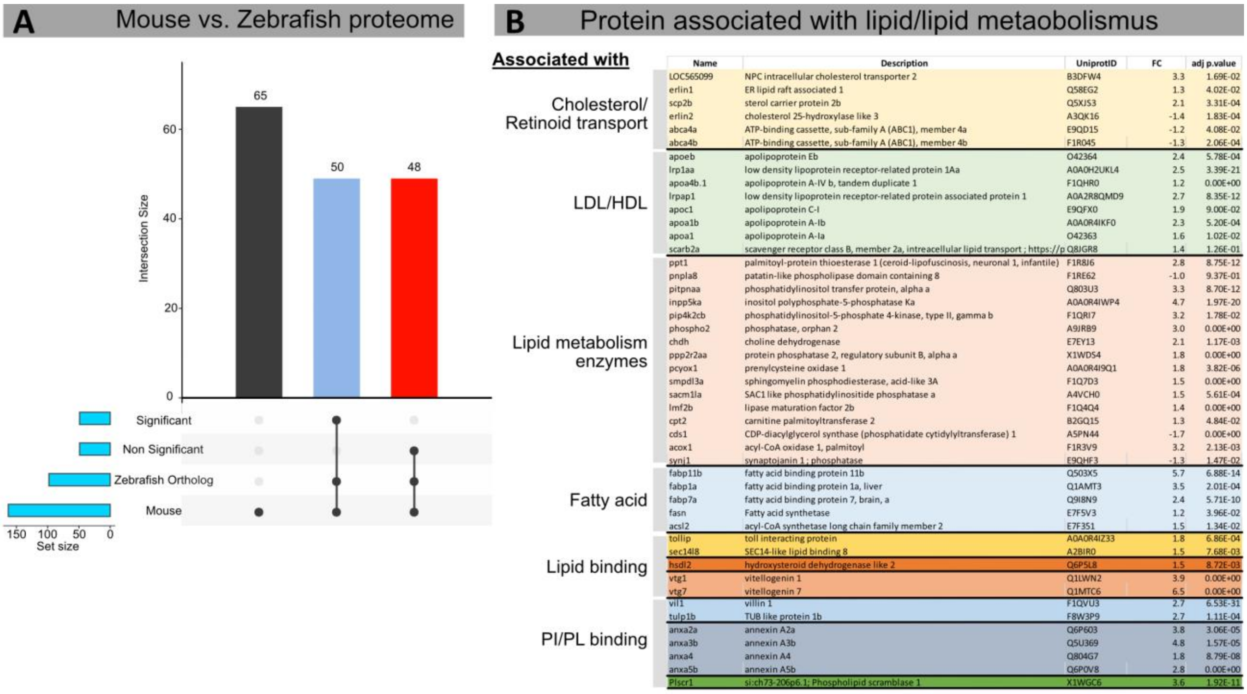

B Protein associated with lipid/lipid metaobolismus

Associated with	Name	Description	UniprotID	FC	adj p.value
Cholesterol/ Retinoid transport	LOC565099	NPC intracellular cholesterol transporter 2	B3DFW4	3.3	1.69E-02
	erlin1	ER lipid raft associated 1	Q58EG2	1.3	4.02E-02
	scp2b	sterol carrier protein 2b	Q5XJS3	2.1	3.31E-04
	erlin2	cholesterol 25-hydroxylase like 3	A3QK16	-1.4	1.83E-04
	abca4a	ATP-binding cassette, sub-family A (ABC1), member 4a	E9QD15	-1.2	4.08E-02
	abca4b	ATP-binding cassette, sub-family A (ABC1), member 4b	F1R045	-1.3	2.06E-04
LDL/HDL	apoeb	apolipoprotein Eb	O42364	2.4	5.78E-04
	lrp1aa	low density lipoprotein receptor-related protein 1Aa	A0A0H2UKL4	2.5	3.39E-21
	apoa4b.1	apolipoprotein A-IV b, tandem duplicate 1	F1QHR0	1.2	0.00E+00
	lrpap1	low density lipoprotein receptor-related protein associated protein 1	A0A2R8QMD9	2.7	8.35E-12
	apoc1	apolipoprotein C-I	E9QFX0	1.9	9.00E-02
	apoa1b	apolipoprotein A-Ib	A0A0R4IKF0	2.3	5.20E-04
	apoa1	apolipoprotein A-Ia	O42363	1.6	1.02E-02
	scarb2a	scavenger receptor class B, member 2a, intreacellular lipid transport ; https://p	Q8JGR8	1.4	1.26E-01
Lipid metabolism enzymes	ppt1	palmitoyl-protein thioesterase 1 (ceroid-lipofuscinosis, neuronal 1, infantile)	F1R8J6	2.8	8.75E-12
	pnpla8	patatin-like phospholipase domain containing 8	F1RE62	-1.0	9.37E-01
	pitpnaa	phosphatidylinositol transfer protein, alpha a	Q803U3	3.3	8.70E-12
	inpp5ka	inositol polyphosphate-5-phosphatase Ka	A0A0R4IWP4	4.7	1.97E-20
	pip4k2cb	phosphatidylinositol-5-phosphate 4-kinase, type II, gamma b	F1QRI7	3.2	1.78E-02
	phospho2	phosphatase, orphan 2	A9JRB9	3.0	0.00E+00
	chdh	choline dehydrogenase	E7EY13	2.1	1.17E-03
	ppp2r2aa	protein phosphatase 2, regulatory subunit B, alpha a	X1WD54	1.8	0.00E+00
	pcyox1	prenylcysteine oxidase 1	A0A0R4I9Q1	1.8	3.82E-06
	smpdl3a	sphingomyelin phosphodiesterase, acid-like 3A	F1Q7D3	1.5	0.00E+00
	sacm1la	SAC1 like phosphatidylinositide phosphatase a	A4VCH0	1.5	5.61E-04
	lmf2b	lipase maturation factor 2b	F1Q4Q4	1.4	0.00E+00
	cpt2	carnitine palmitoyltransferase 2	B2GQ15	1.3	4.84E-02
	cds1	CDP-diacylglycerol synthase (phosphatidate cytidylyltransferase) 1	A5PN44	-1.7	0.00E+00
	acox1	acyl-CoA oxidase 1, palmitoyl	F1R3V9	3.2	2.13E-03
	synj1	synaptojanin 1 ; phosphatase	E9QHF3	-1.3	1.47E-02
Fatty acid	fabp11b	fatty acid binding protein 11b	Q503X5	5.7	6.88E-14
	fabp1a	fatty acid binding protein 1a, liver	Q1AMT3	3.5	2.01E-04
	fabp7a	fatty acid binding protein 7, brain, a	Q9I8N9	2.4	5.71E-10
	fasn	Fatty acid synthetase	E7F5V3	1.2	3.96E-02
	acsl2	acyl-CoA synthetase long chain family member 2	E7F351	1.5	1.34E-02
Lipid binding	tollip	toll interacting protein	A0A0R4IZ33	1.8	6.86E-04
	sec14l8	SEC14-like lipid binding 8	A2BIR0	1.5	7.68E-03
	hsdl2	hydroxysteroid dehydrogenase like 2	Q6P5L8	1.5	8.72E-03
	vtg1	vitellogenin 1	Q1LWN2	3.9	0.00E+00
	vtg7	vitellogenin 7	Q1MTC6	6.5	0.00E+00
PI/PL binding	vil1	villin 1	F1QVU3	2.7	6.53E-31
	tulp1b	TUB like protein 1b	F8W3P9	2.7	1.11E-04
	anxa2a	annexin A2a	Q6P603	3.8	3.06E-05
	anxa3b	annexin A3b	Q5U369	4.8	1.57E-05
	anxa4	annexin A4	Q804G7	1.8	8.79E-08
	anxa5b	annexin A5b	Q6P0V8	2.8	0.00E+00
	Plscr1	si:ch73-206p6.1; Phospholipid scramblase 1	X1WGC6	3.6	1.92E-11

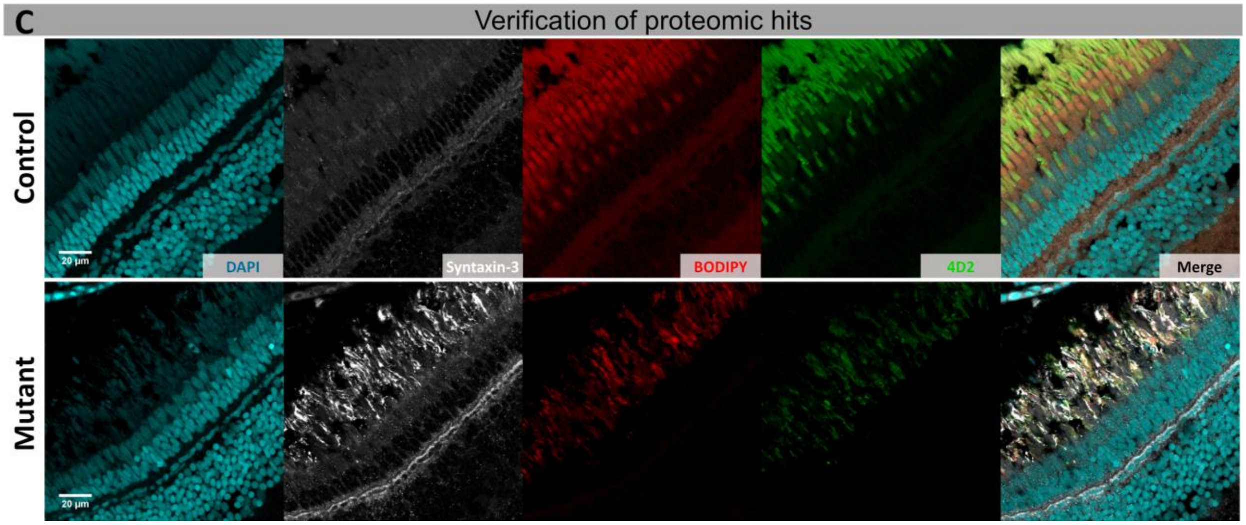

(A) Up-set plot showing the direct comparison between the outer segment proteome of *Bbs17* mutant mouse (Datta et al 2015) and our *bbs1* zebrafish dataset. Of the 146 proteins that were significantly enriched in the *Bbs17* mouse OSs, we found 98 zebrafish proteins that are orthologues to 81 mouse proteins (due to the additional genome duplication in teleosts, in some cases we found several zebrafish orthologues for a single mouse protein). Of the 81 mouse proteins, we found 45 orthologous zebrafish proteins to be also significantly differently expressed. For 65 mouse proteins, no zebrafish orthologue was present in our data set in controls or mutants. The comparison demonstrates a substantial overlap between our dataset and the dataset from *Bbs17* mouse, supporting a conserved role for the BBSome between mouse and zebrafish and validating the findings from our zebrafish dataset (see Supplementary data 4). (**B**) Table describing the large number of lipid-associated proteins that were found to be enriched in the zebrafish *bbs1* mutant OS sample. We listed all the proteins found and grouped them according to

their properties or function. Linear mixed effect models were used to estimate fold changes and P-values, which are adjusted for multiple testing by the Benjamini-Hochberg procedure. Details on the statistical testing of the proteomic data can be found in the methods section. (**C**) Immunohistochemistry staining of 5-month-old retinal sections confirms the increased syntaxin-3 (white) and decreased opsin (4D2, green) in mutant OSs that were observed by the label free quantitative proteomics. Sections are counter-stained using DAPI (nucleus-cyan) and membranes using BODIPY (red). Scale bar: 20μM.

Supplementary Fig. 12: Over-representation analysis of the proteomics dataset

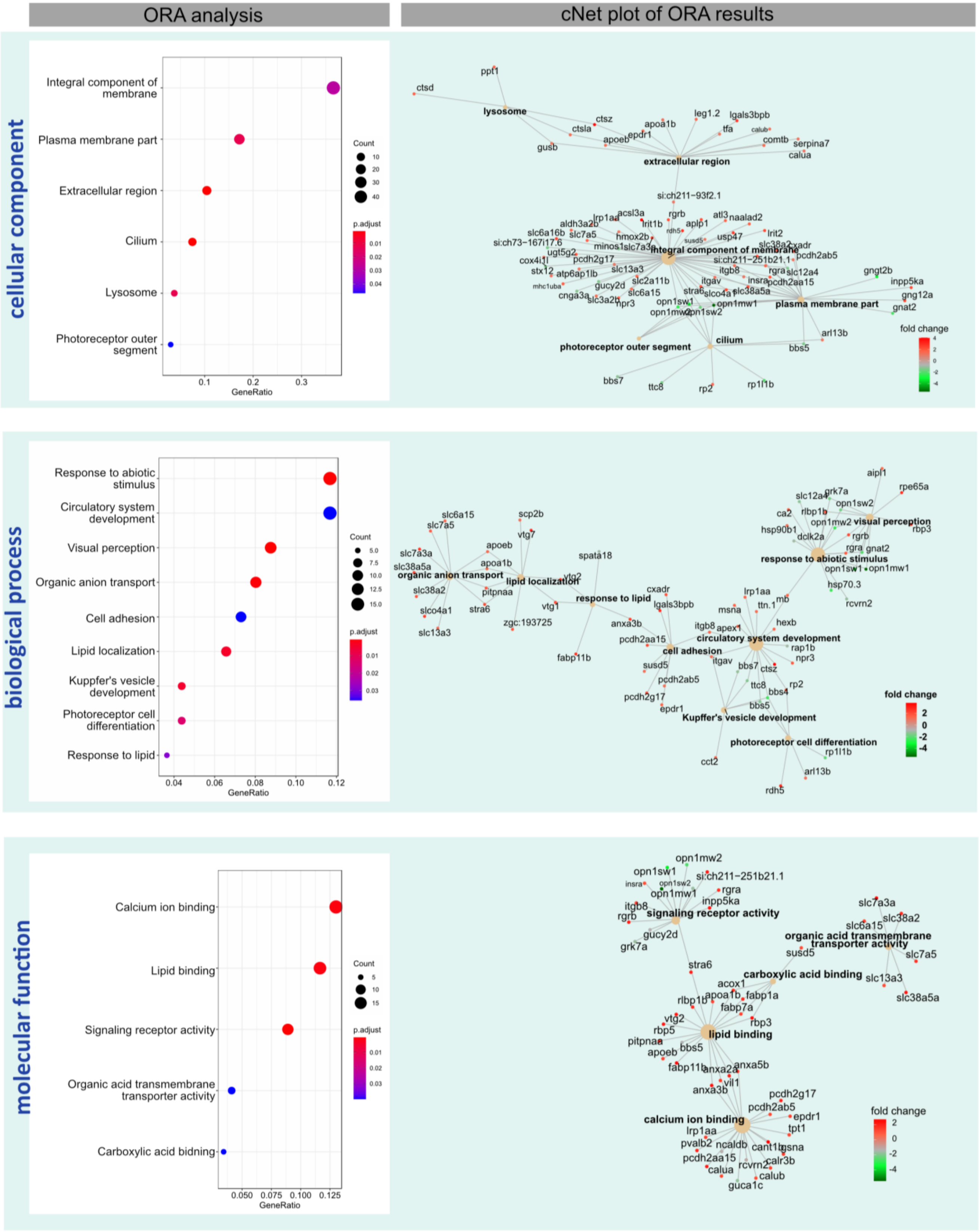

Over-representation analysis of all significant GO-terms found in the cellular component (top), biological process (middle) and molecular function (bottom) using the proteomic dataset (adj. P-val <0.05 & FC>±2). P-values are calculated by hypergeometric distribution and are adjusted for multiple comparison following the Benjamini-Hochberg procedure. Terms that are significantly over-represented are shown in the dot plot (**left plots**). cNet plots (**right plots**) show the network of genes annotated to an over-represented GO-term. Details on the statistics are found in the methods section.

Supplementary Fig. 13: Photoreceptors but not RPE cells express ApoE at 6 dpf

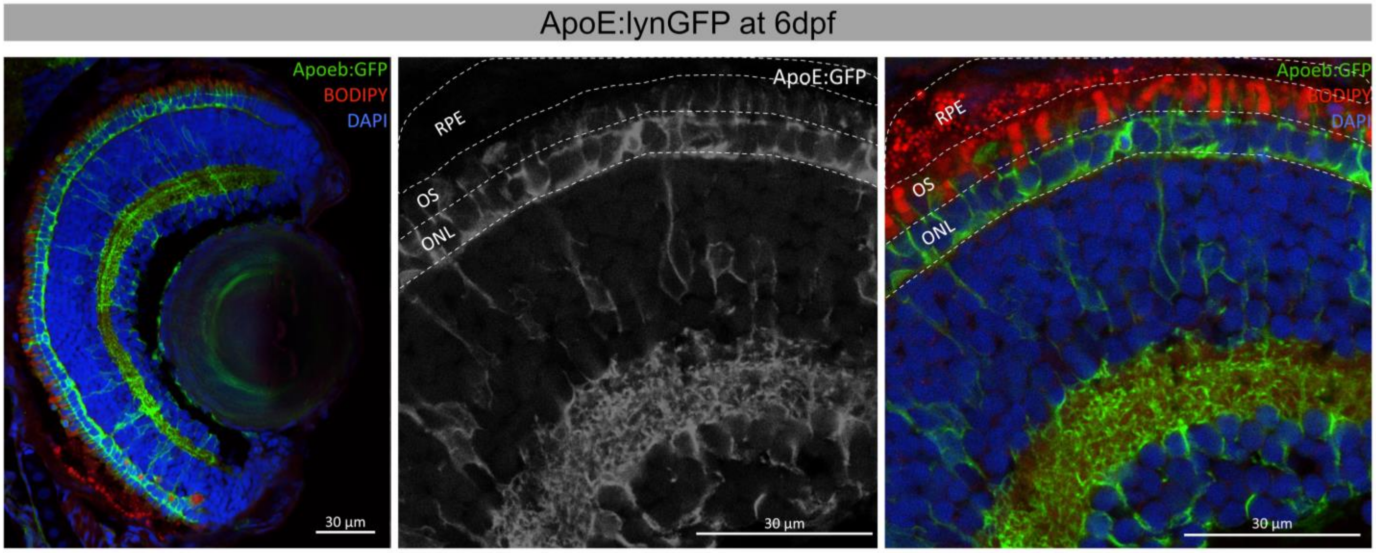

ApoE:lynGFP expression in a retinal section of a 6 dpf transgenic fish. GFP signal is found in photoreceptors and most likely Müller glia cells, but not in RPE cells. Scale bar: 30 µm; Abbreviations: *OS*, Outer segment; *RPE*, retinal pigment epithelium, *ONL* outer nuclear layer.

Supplementary Fig. 14: Total serum cholesterol is unaffected in adult *zgbbs1* mutants.

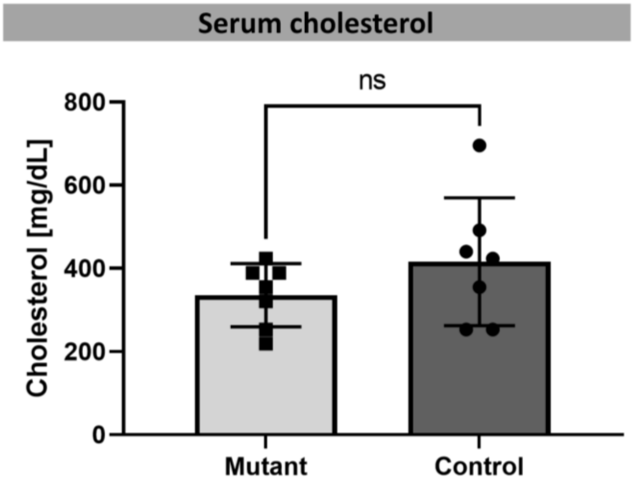

The blood serum of 5-month old *zgbbs1* mutant fish shows no increase in total cholesterol levels compared to age matched wild-type controls (n=7 control fish, n=7 mutant fish, independent two-tailed t-test, P=0.2385, ns not significant, error bars show the standard deviation around the mean). This indicates that the cholesterol accumulation in the eye originates from a local distribution defect and not from systemic hypercholesterolemia.

Source Images:

Suppl. Fig 3b

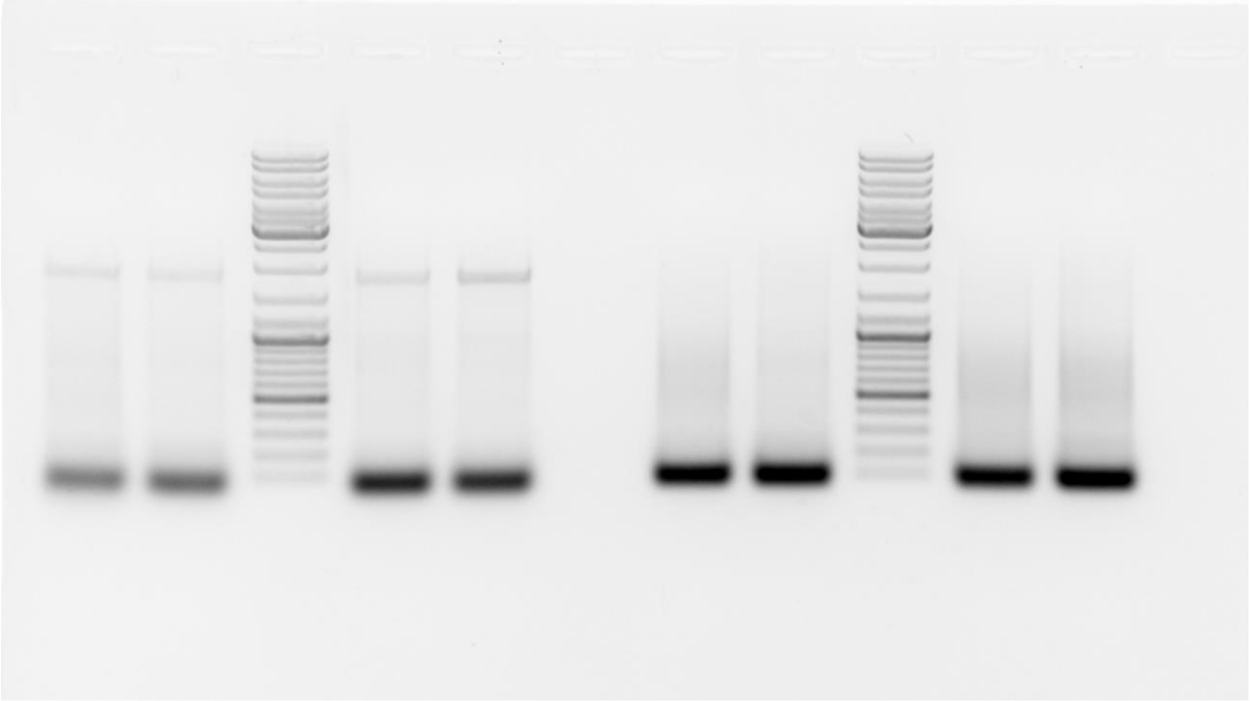

Uncropped image of RT-PCR on adult eye and testis: right hand gel is the no-DNA control showing the primer dimer bands; left gel shows the experiment including control (left) and mutant (right) DNA. No additional bands are visible beyond the band of interest and the primer dimer band.
