# Supplementary material for: Loss of the Bardet-Biedl protein Bbs1 alters photoreceptor outer segment protein and lipid composition
Source: Nat Commun. 2022 Mar 11;13:1282. doi: 10.1038/s41467-022-28982-6 (PMC8917222; doi:10.1038/s41467-022-28982-6)
Supplement: Supplementary file 3 — Description of Additional Supplementary Files [file 41467_2022_28982_MOESM3_ESM.pdf]

Description of Additional Supplementary Files

File Name: Supplementary Data 1
Description: **Whole_adult_eye_proteomics**
Table of all detected proteins in whole adult eye lysates showing MAXquant intensities and differential abundance results. For details on the statistics, please consult methods section.

File Name: Supplementary Data 2
Description: **RNAseq_Dataset**
Table of all genes found in the transcriptomic investigation with their normalized read counts and different expression analysis by Deseq2. The Benjamini-Hochberg adjusted P-values from the Wald test are reported along the P-values

File Name: Supplementary Data 3
Description: **Proteomics_Dataset**
Table of all detected proteins containing MAXquant intensities and different expression results. Fold changes and P-values were estimated form the linear mixed-effects model and the P-values were corrected for multiple testing, using the Benjamini-Hochberg procedure. Details about the statistics can be found in the methods section.

File Name: Supplementary Data 4
Description: **Dre_bbs1-/-_vs_Bbs17-/-_mouse_OS_Proteome**
Table showing the direct comparison of the OS proteomic results from the zebrafish bbs1 dataset presented in this work with the mouse Bbs17 mutant dataset published by Datta et al. 2015. Details about the statistics can be found in the methods section.

File Name: Supplementary Data 5
Description: **Lipidomic_Dataset**
Table of all detected lipids of 5 month old mutant OSs. Raw data and processed data are shown in different worksheets. MetaboAnalyst provides fold change analysis and P-values following a two-sided t-test statistics. Details about the statistics can be found in the methods section.

File Name: Supplementary Data 6
Description: **Detailed statistics_ significance values**
Table of the precise P-values, degrees of freedom, T-ratios and q-values of each multiple T-test.
