# Supplementary material for: Loss of the Bardet-Biedl protein Bbs1 alters photoreceptor outer segment protein and lipid composition
Source: Nat Commun. 2022 Mar 11;13:1282. doi: 10.1038/s41467-022-28982-6 (PMC8917222; doi:10.1038/s41467-022-28982-6)
Supplement: Supplementary file 10 — Reporting Summary [file 41467_2022_28982_MOESM10_ESM.pdf]

nature portfolio

Corresponding author(s): Ruxandra Bachmann-Gagescu

Last updated by author(s): 2022.02.04

Reporting Summary

Nature Portfolio wishes to improve the reproducibility of the work that we publish. This form provides structure for consistency and transparency in reporting. For further information on Nature Portfolio policies, see our Editorial Policies and the Editorial Policy Checklist.

Statistics

For all statistical analyses, confirm that the following items are present in the figure legend, table legend, main text, or Methods section.

n/a	Confirmed	
☐	☒	The exact sample size (*n*) for each experimental group/condition, given as a discrete number and unit of measurement
☐	☒	A statement on whether measurements were taken from distinct samples or whether the same sample was measured repeatedly
☐	☒	The statistical test(s) used AND whether they are one- or two-sided<br>*Only common tests should be described solely by name; describe more complex techniques in the Methods section.*
☒	☐	A description of all covariates tested
☐	☒	A description of any assumptions or corrections, such as tests of normality and adjustment for multiple comparisons
☐	☒	A full description of the statistical parameters including central tendency (e.g. means) or other basic estimates (e.g. regression coefficient) AND variation (e.g. standard deviation) or associated estimates of uncertainty (e.g. confidence intervals)
☐	☒	For null hypothesis testing, the test statistic (e.g. *F*, *t*, *r*) with confidence intervals, effect sizes, degrees of freedom and *P* value noted<br>*Give P values as exact values whenever suitable.*
☒	☐	For Bayesian analysis, information on the choice of priors and Markov chain Monte Carlo settings
☒	☐	For hierarchical and complex designs, identification of the appropriate level for tests and full reporting of outcomes
☐	☒	Estimates of effect sizes (e.g. Cohen's *d*, Pearson's *r*), indicating how they were calculated

Our web collection on statistics for biologists contains articles on many of the points above.

Software and code

Policy information about availability of computer code

Data collection	ImageJ (vers. 1.53c)
Data analysis	R with the package: Deseq2 (vers. 1.34.0), ClusterProfiler(vers. 3.10.1) , lmerTest (3.1.0), enrichplot (vers. 1.2.0)<br>UNIX package: featureCount (vers. 2.0.1), SAMtools (1.7),<br>Software: MaxQuant (version 1.6.2.3), Skyline (20.1), GraphPad Prism (vers. 9.0.0), Unipro UGENE (vers. 36.0),<br>Webtools: MetaboAnalyst (vers. 5.0) , WEB-Gestalt

For manuscripts utilizing custom algorithms or software that are central to the research but not yet described in published literature, software must be made available to editors and reviewers. We strongly encourage code deposition in a community repository (e.g. GitHub). See the Nature Portfolio guidelines for submitting code & software for further information.

Data

Policy information about availability of data

All manuscripts must include a data availability statement. This statement should provide the following information, where applicable:

- Accession codes, unique identifiers, or web links for publicly available datasets
- A description of any restrictions on data availability
- For clinical datasets or third party data, please ensure that the statement adheres to our policy

All data generated or analysed during this study are included in this published article (and its Supplementary Information files or source data file). The proteomic data have been deposited in the ProteomeXchange Consortium via the PRIDE [https://www.ebi.ac.uk/pride] partner repository under accession code PXD026646 for the OS proteome and PXD030522 for the whole eye lysate proteome. The RNAseq data have been deposited in the SRA repository under the BioProject accession number PRJNA789116 [https://www.ncbi.nlm.nih.gov/sra]. The lipidomic data have been deposited in the MetaboLights database under accession code

MTBLS4013 [https://www.ebi.ac.uk/metabolights/]. The processed RNAseq, proteomic and lipidomic data are available in the respective supplementary tables. The PRIDE-Database are currently set to private mode (accessible with Username: reviewer_pxd026646@ebi.ac.uk, Password: z1gurkKw) and will be made publicly available upon acceptance. The NCBI/SRA RNAseq data is released and the lipidomic data has been uploaded but is still under curation through the metabolights database.

Field-specific reporting

Please select the one below that is the best fit for your research. If you are not sure, read the appropriate sections before making your selection.

- [x] Life sciences
- [ ] Behavioural & social sciences
- [ ] Ecological, evolutionary & environmental sciences

For a reference copy of the document with all sections, see nature.com/documents/nr-reporting-summary-flat.pdf

Life sciences study design

All studies must disclose on these points even when the disclosure is negative.

Sample size

Sample sizes were chosen based on practical and scientific considerations: for stainings and ERGs on larvae, at least 10 animals per condition were chosen in each experiment; where quantifications were performed, this sample size was sufficient to determine statistical significance. For adults, which are hard to raise as mutant, as many fish as available were used (i.e. for ERG on 3-month old fish). For -omics experiments, we used standard sample sizes (n=3 for transcriptomics or n=4 for proteomics) based on statistical considerations. In these experiments, each sample represented a pool of as many animals as required to allow robust detection by the method employed (e.g. 7 retinae from 4 fish to retrieve sufficient amounts of protein for MS/MS).

Data exclusions

no data were excluded

Replication

All experiments were repeated at least twice with multiple animals from distinct clutches, also representing biological replicates within each experiment (larvae and adults). Relevant variability was reported when observed (e.g. such as body curvature in mutant larvae); no conflicting results were observed (i.e. replication was always successful).

Randomization

No randomization was performed since we have two conditions (control and mutant) which are inherently distinct from each other. Therefore, the genetic background (possessing the mutation or not) defines the allocation into that group. The studies were analyzed/ measured in a blinded manner wherever possible to evade biases.

Blinding

Image analysis was performed in a blinded manner as to genotype. For ERGs, genotyping was performed after the measurement (so the measurement was done without knowledge of the genotype).

Reporting for specific materials, systems and methods

We require information from authors about some types of materials, experimental systems and methods used in many studies. Here, indicate whether each material, system or method listed is relevant to your study. If you are not sure if a list item applies to your research, read the appropriate section before selecting a response.

Materials & experimental systems

n/a	Involved in the study	
[ ]	[x]	Antibodies
[x]	[ ]	Eukaryotic cell lines
[x]	[ ]	Palaeontology and archaeology
[ ]	[x]	Animals and other organisms
[x]	[ ]	Human research participants
[x]	[ ]	Clinical data
[x]	[ ]	Dual use research of concern

Methods

n/a	Involved in the study	
[x]	[ ]	ChIP-seq
[x]	[ ]	Flow cytometry
[x]	[ ]	MRI-based neuroimaging

Antibodies

Antibodies used

Primary antibodies were mouse anti-4D2 (1:200, gift from R. Molday, University of British Colombia), mouse-anti-Zpr-1 (1:200, Zebrafish International Resource Center, Eugene, https://zfin.org/ZDB-ATB-081002-43), rabbit-anti-UV-opsin (1:300, gift from D. Hyde, https://zfin.org/ZDB-ATB-090508-3), mouse-anti-SV2 (1:100, Developmental Studies Hybridoma Bank, https://zfin.org/ZDB-ATB-081201-1), rabbit anti-Arl13b (1:100, gift from Z. Sun, Yale University, https://zfin.org/ZDB-ATB-100113-1), mouse-anti-acetylated-Tubulin (1:500, clone 611B-1, Cat# MABT868, SIGMA), rabbit- anti syntaxin-3 (1:200, Alomone labs, Cat# ARN-005) and mouse monoclonal anti-polyglutamylated tubulin (1:400,GT335 clone, Cat# alx-804-885-c100, Enzo Life Sciences). Secondary antibodies were Alexa Fluor-conjugated goat anti-rabbit (Alexa488, Cat#A-11008) or goat anti-mouse (Alexa488, Cat#A28175; Alexa568, Cat#A-11004) IgG (1:400, Life Technologies, Darmstadt, Germany).

Validation	Anti-4D2: used in several zebrafish publications (e.g. Ojeda Naharros et al, doi: 10.1371/journal.pgen.1007150).<br>Anti-Zpr-1: validated and used in many zebrafish publications [http://zfin.org/ZDB-ATB-081002-43#summary]<br>Anti-UV-opsin: used in several zebrafish publications (https://zfin.org/ZDB-ATB-090508-3#summary).<br>Anti-SV2: validated and used in many zebrafish publications [http://zfin.org/ZDB-ATB-081201-1#summary]<br>Anti Arl13b: used in several zebrafish publications (e.g. Duldulao et al, doi:10.1242/dev.036350).<br>Anti-ac.Tubulin: validated and used in many zebrafish publications [http://zfin.org/ZDB-ATB-081003-6#summary]<br>anti Syntaxin-3: Used in our lab and previously published (e.g. Ojeda Naharros et al, doi: 10.1371/journal.pgen.1007150)<br>Anti- GT335: used in many of our publication and by the zebrafish community [http://zfin.org/ZDB-ATB-090818-1#summary]

Animals and other organisms

Policy information about studies involving animals; ARRIVE guidelines recommended for reporting animal research

Laboratory animals	Zebrafish bbs1 mutant and control were raised in the AB background. Experiments were perforemd in larval stages or adult stage up to 10 months. The sex of zebrafish is not determined at larval stages and therefore not taken into consideration. Adult animals of both sexes were used.
Wild animals	No wild animals were used in the study.
Field-collected samples	No field collected samples were used in the study.
Ethics oversight	Veterinäramt Zürich , Switzerland and Regierungspräsidium, Karlsruhe, Germany

Note that full information on the approval of the study protocol must also be provided in the manuscript.
